# Development of a transition program for emerging adults with type 1 diabetes: A quality improvement initiative

**DOI:** 10.1016/j.hctj.2024.100073

**Published:** 2024-10-14

**Authors:** Elizabeth A. Minchau, Billie S. Vance, Emily Barnes

**Affiliations:** a Department of Adult Health, School of Nursing, West Virginia University (Dr. Minchau), Morgantown, WV, USA; b Department of Family and Community Health, School of Nursing, West Virginia University (Dr. Vance), Morgantown, WV, USA; c School of Nursing, West Virginia University (Dr. Barnes), Morgantown, WV, USA

**Keywords:** Type 1 diabetes, Adolescence, Healthcare transition, Quality improvement

## Abstract

**Background:**

Emerging adults with type 1 diabetes (T1D) struggle with glycemic management that can be exacerbated by a disorganized transition process. Substantial gaps in care have been noted during this transition period and have been shown to lead to suboptimal health care utilization, worsening glycemic management, increased incidence of serious complications, and mental health challenges. A formal transition program can help prevent gaps in care, improve disease self-management skills, decrease disease burden, and reduce diabetes complications.

**Purpose:**

There is an increased focus within the pediatric community to formally prepare young adults with T1D as they transition to the adult health care setting. The purpose of the quality improvement initiative was to develop a transition program for adolescents with T1D in a pediatric endocrinology clinic within an academic medical center.

**Methods/interventions:**

The Institute for Healthcare Improvement *Model for Improvement* was used to guide this project. Got Transition's® *Six Core Elements of Health Care Transition* were adapted to the unique needs of the pediatric endocrinology setting by incorporating diabetes-specific transition guidelines recommended by the American Diabetes Association (ADA). A new program was designed to target patients between the ages of 16–18 offering structured transition visits focused on enhancing knowledge and skills related to the management of T1D and improving the transfer of care process between pediatric and adult providers. Key elements of the program were integrated into the electronic health record. A focus group with clinical partners was used to evaluate the acceptability and practicality of the program.

**Results:**

Transition activity within the clinic increased from 12/32–28/32 on the *Current Assessment of Healthcare Transition Activities* tool. Four themes were identified from clinical partner feedback supporting the acceptability and practicality of program design.

**Conclusions:**

The integration of the Got Transition® framework and the ADA recommendations supports clinicians in meeting the unique needs of adolescents with T1D. Incorporating the transition activities into the electronic health record facilitated integration into the workflow of the clinic providers. This initiative can serve as a model to expand transition activities across pediatric specialty care settings.

## Highlights

• Got Transition® was used to create a transition program, *Preparing Emerging Adults with Knowledge and Skills* (PEAKS), to improve transition practices in pediatric endocrinology.
• The PEAKS program incorporated 14 ADA recommendations for transition and provided educational modules, flowsheet documentation, readiness assessment, and enhanced communication.
• The entire PEAKS program was integrated into the health system's electronic health record.
• During feasibility analysis, the PEAKS program was deemed acceptable and practical by stakeholders.

## 1 Introduction

For many young adults with chronic disease, the transition from the pediatric to the adult healthcare setting is not well planned and often results in gaps in care, preventable complications, and avoidable costs.^1^, ^2^ There is a growing recognition of the need to support vulnerable adolescents with type 1 diabetes (T1D) as they transition to new adult providers.^3^, ^4^ Organizing the transition process for adolescents with chronic conditions, including T1D, is imperative to enhance continuity of care, improve treatment adherence, reduce morbidity, increase patient and family satisfaction, and prevent emergency room and hospital visits.^5^ Formal healthcare transition processes offer resources for self-management, service coordination, and referral to community-based services; place emphasis on fostering independence, self-efficacy, and the developmental competencies required in adulthood; and focus on the emerging adult and those who support them in their daily care.^6^, ^7^

### 1.1 Problem description

Creating effective and translatable pathways for the transition of care from pediatric to adult healthcare settings helps to decrease risk, enhance disease self-management, and optimize well-being.^8^, ^10^, ^11^ However, the implementation of organized transition pathways remains inadequate within the United States healthcare system^11^ due to the time and resources needed to successfully implement the transition process.^3^ Given the organizational changes and evolving resources and priorities that occurred with the opening of a brand-new free-standing Children’s Hospital within a large healthcare system, a renewed commitment to improving transitional care surfaced and served as the impetus for the quality improvement initiative described in this manuscript.

### 1.2 Available knowledge

It is estimated that 304,000 children and adolescents younger than age 20 are diagnosed with T1D in the United States.^12^ The prevalence of T1D in children and young adults worldwide has doubled in the past 25 years and is expected to double yet again in the next 15–20 years.^13^, ^14^, ^15^ The management of complex chronic conditions, like TID, can pose particular challenges for the transition-aged population. The typical developmental challenges that occur during the transition time period are amplified in emerging adults with T1D who demonstrate heightened vulnerability as they assume responsibility for their chronic illness, encounter significant change in daily routine and parent/caregiver involvement, and potentially begin to engage in high-risk behaviors.^16^

The developmental stage of adolescence and young adulthood encompasses elements of physical change and major social role transitions and often coincides with other significant life events, such as a move to college or into the workforce, separation from family of origin, and a change in geographic location or place of residence. Adolescents tend to be under-prepared to independently navigate the required self-care responsibilities that are common with progression into young adulthood.^3^ Pursuing developmentally appropriate educational, occupational, and social activities often take priority over management of chronic disease. Thus, it is not surprising that self-care behaviors, disease control, routine screenings, and medical follow-up are not prioritized in young adults who feel they lack the time, support, understanding, skills, and partnerships necessary to successfully manage their disease and make treatment decisions independently.^17^ This results in gaps in diabetes care, such as missed healthcare visits and sub-optimal diabetes self-management related to self-monitoring and daily medication administration and can lead to deterioration of glycemic management, development of acute complications, emergence of chronic complications that may go undetected or untreated, and social, financial, behavioral, and psychological challenges.^8^ The rapidly increasing prevalence of T1D, coupled with the developmental challenges occurring in children and adolescents, highlights the pressing need to address the health demands of this patient population as they transition from pediatric to adult care.

Transition interventions can be highly variable.^18^ Initiatives that target young adults with T1D through education, skill enhancement, specialty transition clinic or focus, and/or official transition coordinator roles, yield positive outcomes.^18^ As programs are designed, they need to remain focused on the developmental complexities of young adulthood, the multi-faceted cognitive, psychomotor, social, and affective requirements of successful diabetes management, and the feasibility of program implementation.^8^, ^17^, ^18^, ^19^

### 1.3 Rationale

The need for structured transition has been acknowledged by the American Academy of Pediatrics (AAP), the American Academy of Family Physicians (AAFP), the American College of Physicians (ACP), and the American Diabetes Association (ADA).^8^, ^9^ Formalized transition processes help bridge the gap between dependent care with parental management and oversight and independent disease management with a focus on self-care, self-efficacy, enhanced disease knowledge and disease decision-making, and continuity of care.^8^, ^17^, ^18^, ^19^ The National Alliance to Advance Adolescent Health’s Got Transition® program can be utilized as a guide to develop transition programs for young adults with chronic disease as they transition from pediatric to adult healthcare.^20^ Got Transition® utilizes the *Six Core Elements of Healthcare Transition*, which outline the essential components of a structured transition process. These elements can be tailored to the specific setting facilitating the healthcare transition. The Got Transition® approach has been adapted to a variety of clinical settings and can facilitate early implementation of healthcare transition improvement efforts.^5^

### 1.4 Specific Aims

This paper describes the creation of a transition program in a pediatric endocrinology clinic. The specific objectives that guided program development were to adapt the Got Transition® framework^20^ to the specific needs of the outpatient pediatric endocrinology clinic and to examine the feasibility of the program.

## 2 Methods

### 2.1 Context and clinical partners

The setting for this quality improvement project was an outpatient pediatric endocrinology clinic which is part of an academic-medical center in the southeast region of the United States. It is the only pediatric endocrinology clinic in a 90-mile radius and serves children and adolescents (ages 0–18 years old) with T1D from a multi-state area. An average of 20 patient encounters occur during each of the three clinic days per week that are reserved for diabetes management. Clinic appointments are typically between 20 and 30 minutes long with time split between a medical provider (physician) and a diabetes educator (nurse or dietician). Epic is the electronic health record (EHR) utilized by the healthcare system. Key clinical partners involved in this project included healthcare providers and diabetes educators who work within the clinic. These partners contributed input during all phases of program design and implementation. Informal partners, including patients and their families/caregivers, and adult healthcare providers, also contributed ideas related to program needs during informal discussions occurring in the weeks prior to program development. Established patients, families, and caregivers were asked specifically for their insight related to overall transition needs, priority education topics, and preferred timing of program integration and delivery. Endocrinology providers within the academic medical center who manage adult patients with diabetes were asked to provide perspective related to common knowledge deficits they encounter in new patients with T1D who have transitioned from pediatric care.

### 2.2 Interventions

#### 2.2.1 Transition Program

**Fig. 1:**
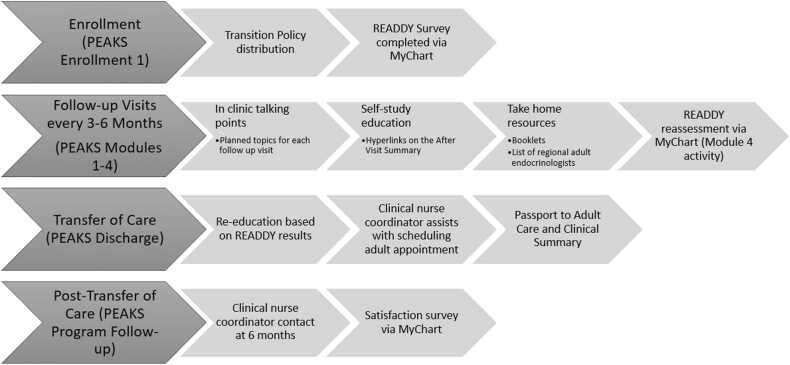
PEAKS Program Progression. *This illustrates the progression through the various PEAKS program modules.*

The Six Core Elements of Health Care Transition™ 3.0^21^ and the 14 Recommendations for Transition from the ADA^8^ guided program development (see Table 1). The transition program, *Preparing Emerging Adults with Knowledge and Skills* (PEAKS), was designed for adolescents with T1D between the ages of 16–18 (Fig. 1). Due to the frequency of patient appointments in diabetes clinic (which typically occur every 3 months), this two-year time frame was identified by clinical partners as ideal, as well as feasible, for focused transition preparation. The entire PEAKS program was integrated into Epic.

**Table 1:** Integration of Six Core Elements and ADA Recommendations into PEAKS Program.

**Six Core Elements of HCT**	**ADA Practice Guideline**	**PEAKS Program Design**
**Transition Policy**	Sharing purpose, goals and objectives; orienting patients to adult care; intervening with respect to developmental age	Policy development and distribution; enrollment at age 16; two-year design with scaffolded education every 3 months
**Tracking/Monitoring**	Closing gaps in care; screening for eating and affective disorders; screening for complications	Epic Express Lane for automatic enrollment; in-clinic education topics with additional online resources; PEAKS flowsheet for program element documentation
**Transition Readiness**	Self-management; transition topic education; individualized care	READDY assessment using MyChart (given before and after program); clinic talking points using teach-back techniques; after visit summary hyperlinks for continued self-guided learning
**Transition Planning**	Education; communication; goals and action steps	Self-management education and application at each visit; offer list of adult providers; focus on personal goal setting
**Transfer of Care**	Aligning adult endocrinology care and primary care; use of a written clinical summary	Complete *Passport to Adult Care* and *Clinical Summary*
**Transfer Completion**	Making first adult appointment for patient; providing close follow-up	Schedule first adult appointment before patient is discharged; follow-up to assure no gaps in care; assess satisfaction with transition process via feedback surveys

#### 2.2.2 Transition Needs Assessment

A baseline assessment of the clinic’s current transition practices was performed using the Current Assessment of Healthcare Transition Activities (CAHTA) tool (https://www.gottransition.org/6ce/?leaving-current-assessment). The tool is designed to evaluate the progress towards incorporating the Six Core Elements of Health Care Transition, and parent/caregiver and youth/young adult feedback and leadership. Each of the eight sections on the tool has criteria which can be assigned a level of 1–4. To achieve a specific level designation in any section, all criteria within the level must be met. A level 4 designation in all sections equates to full implementation of evidence-based transition processes. A baseline score of 12/32 was recorded for the pediatric endocrinology clinic.

#### 2.2.3 Change strategies

The Institute for Healthcare Improvement's Model for Improvement^22^ and plan, do, study, act (PDSA) cycle guided program development, which occurred across four phases. Phases 1 and 2 reflected the "planning" and "doing" stages of the PDSA cycle. Phase 3 served as the "study" stage, and phase 4 reflected the "act" stage. The following paragraphs describe the process of program development and demonstrate how the PDSA cycle guided program design.

##### 2.2.3.1 Phase 1: Early Program Design

After gathering feedback from clinical partners (2 physicians and 2 diabetic educators) on how the ADA recommendations and Six Core Elements of Transition might be adapted to the clinic’s unique needs, program design began. A blueprint of all the elements necessary to create the program was developed (see Table 1). The first element developed was a transition policy that described the clinic’s position on transition and rationale for the PEAKS program. The second element developed was a flowsheet for tracking and monitoring. The third element included selection of a readiness assessment tool. Clinic partners decided on the Readiness of Emerging Adults with Diabetes Diagnosed in Youth (READDY) survey, which is a diabetes-specific transition readiness assessment tool used to identify educational topic areas in which the patient recognizes a need for more knowledge.^23^ The READDY survey includes four domains: diabetes knowledge, health system navigation, insulin self-management, and health behaviors. Additionally, general education topics were identified, prioritized, and grouped into four separate modules based on feedback from clinical partners (see Table 2). Each module was intended to be delivered across follow up visits throughout the duration of the program. In response to clinical partner feedback, a desk-copy of “talking points” (educational topics) related to each module's focused learning themes was provided (see Table 2). The final three elements included development of communication tools for patients and providers ("passport to adult care" and "clinical summary"), a list of regional adult providers, and a plan for 6-month follow up. The plan for follow up was designed for the clinic’s existing nurse coordinator to contact the patient at 6 months via phone and offer a transition satisfaction survey. As elements of program design were proposed, clinical partners continued to prioritize the need for an integrated program that improved flow and organization without causing additional time-burden.

**Table 2:** Education Topics and AVS Hyperlinks.

	**Topics**	**Hyperlinks attached to After Visit Summary**	**Resources**
Module 1	1. Driving with diabetes 2. Glucagon administration (including demonstration) 3. Mental health and diabetes distress	• Hyperlink 1: Driving with Diabetes: https://www.joslin.org/patient-care/diabetes-education/diabetes-learning-center/5-tips-remember-when-you-get-behind-wheel and https://beyondtype1.org/the-driving-diabetic/ • Hyperlink 2: Glucagon administration: https://beyondtype1.org/glucagon/ • Hyperlink 3: Mental Health and Diabetes Distress: https://beyondtype1.org/mental-health/	
Module 2	4. Medical alert bracelets or medical ID features on smart phones 5. Exercising with diabetes 6. Food and diet during the teen years	• Hyperlink 4: Medical ID feature on smart phones: https://gottransition.org/resource/?setting-up-medical-id-smartphones • Hyperlink 5: Exercise and Diabetes: https://beyondtype1.org/diabetes-and-exercise/ • Hyperlink 6: Food and Diabetes: https://beyondtype1.org/food-and-diabetes/	
Module 3	7. Independent living with diabetes 8. Plans for sick days 9. Going to college and entering the workforce	• Hyperlink 7: Living independently with diabetes: https://www.jdrf.org/wp-content/uploads/2022/11/JDRF_Living_Independently_with_T1D.pdf • Hyperlink 8: Managing sick days: https://www.cdc.gov/diabetes/managing/flu-sick-days.html • Hyperlink 9: College and diabetes: https://collegediabetesnetwork.org/node/12650	Booklets: • *Off to College with Diabetes* • *Off to Work with Diabetes*
Module 4	10. Differences between adult and pediatric service models 11. Sexuality and reproductive health, drugs, tobacco and alcohol and diabetes 12. Navigating insurance	• Hyperlink 10: Differences between pediatric and adult care: https://gottransition.org/resource/?system-differences-between-pediatric-and-adult-health-care • Hyperlink 11: Sex, drugs, alcohol and tobacco use: https://beyondtype1.org/sex-drugs-diabetes/ • Hyperlink 12: Navigating insurance: https://www.jdrf.org/t1d-resources/living-with-t1d/insurance/	• List of Regional Adult Providers

##### 2.2.3.2 Phase 2: Integration into Electronic Health Record

The blueprint of the transition program was then built into the EHR for efficient integration into daily clinic activities. The capabilities of the EHR were leveraged to allow for automation and templating of program elements to reduce time burden (see Table 3). This included creating an "Express Lane" that opened automatically at the first visit after the 16th birthday. The Express Lane allowed for assignment of the READDY survey to the patient through the EHR patient-portal, population of a PEAKS program flowsheet for tracking program progress, links for documentation smart-phrases, dissemination of educational hyperlinks (see Table 2), and pre-determined billing codes and follow up visit interval.

**Table 3:** Custom Epic Build.

**Custom Builds**	**Explanation**
Express Lane	Allows for automatic enrollment in PEAKS following 16th birthday. Feature to increase provider efficiency by allowing review of key information, order placement, and encounter documentation from a single screen. For instance, the PEAKS Express Lane Enrollment includes a note template, an automated billing code, prepopulated 3-month return appointment, and the transition policy attachment for the AVS. PEAKS Express Lane Module 1 includes a note template, an automated billing code, prepopulated 3-month return appointment, and the first 3 self-study education hyperlinks.
Program Flowsheet	Allows for concise documentation of module and program progress; increases visibility for all providers.
READDY assessment	Diabetes-specific assessment related to readiness for transition to adult care; delivered at enrollment and at PEAKS Module 4 visit; built as a survey in the screening tab
Smart phases	User generated phrases that provide a shortcut for documentation elements and can be added to encounter notes by a provider. For instance, a smart phrase related to each PEAKS Module was created for the diabetic educators.
Note template	Preformatted template that includes common elements needed for documentation of a health care encounter. For PEAKS, the current provider endocrinology visit note was modified to facilitate inclusion of components of PEAKS encounters.
After Visit Summary	Customized for each PEAKS encounter to include appropriate education links, resources, or information. For instance, the PEAKS Transfer of Care encounter AVS included a completed Passport to Adult Care and a Clinical Summary.
Clinical Summary for Transition	Intended to be a provider-to-provider information hand-off. The summary includes the most current medications and dosing, labs values (HgbA1c, lipid panel, TSH, and microalbumin/creatinine ratio), and anticipated patient needs.
Passport to Adult Care	The document includes date, time, and location of 1st adult appointment, a checklist of what to take (i.e., insurance cards, pharmacy name and phone number) to the first appointment, and what to expect at the visit.

##### 2.2.3.3 Phase 3: Staff Trial of Built Program

After integration into the EHR, clinical partners were able to trial the program with simulated patients in the Epic Playground - a simulation platform within Epic. Two providers, two diabetic educators, and two medical staff (medical assistant and LPN) were involved in three patient simulations. This allowed for a preliminary assessment of the function and workflow of the built program. Feedback was solicited from these partners following this trial. Revisions were made to address identified clinical partner concerns. For example, physician providers were concerned that the automatic note template would limit their use of the forward note option, a mechanism within the EHR that allows use of the previous visit's note to serve as an outline for the current visit's note. To address this, documentation options within the EHR were created to allow providers flexibility to embed pertinent PEAKS module visit information into a note template of their choice.

##### 2.2.3.4 Phase 4: Roll-out of Program

After the study phase was completed, the PEAKS program was fully implemented into patient care*.* In addition to patients being enrolled at age 16, established patients older than 16 were also included in the program roll-out. These older patients were offered “catch-up” visits to provide the same opportunity to develop knowledge and skills prior to clinic discharge at age 18. The “catch up” visits required that multiple PEAKS modules be completed in a fewer number of clinic visits, rather than across the program's planned 2-year timeframe. After 20 patient visits, including usual and "catch up" visits, feedback was solicited from clinical partners. Revisions were made based on this feedback. For example, a deferral option was added to allow providers to adjust the PEAKS program progression plan during a follow-up visit should a critical health issue be more pressing.

### 2.3 Measures

Following the IHI model, outcome, process, and balancing measures were identified. The outcome measure was an assessment of the clinic’s transition activities via the CAHTA tool. As noted above, the tool was used to establish a baseline score prior to program development and then used again prior to program roll-out to evaluate progress towards improved transition practices.

The process and balancing measures identified for this project were program practicality (process) and program acceptability (balancing). Both are measures of feasibility.^24^ Practicality is defined as to what extent a program can be carried out with intended participants with the existing means, resources, and circumstances and without outside intervention. Acceptability is to what extent a program is judged as suitable for program deliverers.^24^ To assess practicality and acceptability, a focus group was conducted with our clinical partners (who acted as key informants) using a semi-structured interview guide. The semi-structured guide included 16 open-ended interview questions. The interview questions were designed to elicit feedback about specific outcomes of interest including factors affecting implementation of the PEAKS program, the perceived efficiency, speed, and quality of implementation of the different components of the PEAKS program, the positive/negative effects on target participants, and the ability of participants to carry out intervention activities as currently designed (practicality). In addition, there were questions related to the providers' satisfaction with the program, their intent to continue use of the PEAKS program, and perceived appropriateness of the program (acceptability). The focus group interview was conducted after 10 weeks of patient encounters and included five clinical partners (dietician/diabetic educator (1), nurse coordinator/diabetic educator (1), pediatric endocrinologists (2), and psychology intern (1)). Interviews were audiotaped and transcribed.

### 2.4 Analysis

Overall assessment of transition activities involved tallying the scores on the CAHTA tool and comparing the difference at baseline and at project completion. The transcribed interviews related to program acceptability and practicality were reviewed to evaluate feasibility of program design. Similar text was grouped into categories and themes were identified.

### 2.5 Ethical Considerations

Prior to implementation, the project was reviewed by the Institution’s Office of Human Research Protections and acknowledged as not human subjects research.

## 3 Results

### 3.1 Outcome measures

The final PEAKS program design incorporated all Six Core Elements of Health Care Transition.^21^ In addition, the program design integrated all 14 transition recommendations from the ADA. ^8^ The clinic’s overall transition activity assessment score increased from a 12/32 to a 28/32 on the CAHTA tool from pre-program to post-program implementation over a 6-month time period. The areas in which additional opportunities remain include Core Element 3: *Transition Readiness* and Core Element 6: *Youth/Young Adult and Parent/Caregiver Leadership*. With regard to Core Element 3, *Transition Readiness,* all required criteria to achieve the level 4 designation were met with the exception of a provision of independent time (without parent/caregiver) with the provider beginning between the ages of 12 and 14. The PEAKS program is designed for independent time with the provider to begin at age 16. With regard to Core Element 4, *Youth/Young Adult and Parent/Caregiver Leadership*, there is a plan for the clinic to gather feedback related to satisfaction with the new transition process, but a transition advisory panel that includes parents/caregivers and/or youth/young adults who have recently graduated from the PEAKS program has yet to be developed. Furthermore, a peer support initiative has not yet been instituted and a pathway does not currently exist allowing program graduates to aid in the continuing education of medical providers.

### 3.2 Process measure

Semi-structured interviews with clinical partners identified three themes related to practicality of program design: efficiency of EHR integration, time burden, and identification of knowledge gaps.

#### 3.2.1 Efficiency of EHR integration

Clinical partners appreciated the organized and streamlined integration of program elements into the existing EHR. They provided positive feedback related to the provision of smart-phrases, the READDY transition survey, the in-clinic talking points, and the clinical summary tool. One partner commented on ease of use during the encounter stating, *"*[It is] *impressive how it has been organized and put into Epic and all the elements are pulled in automatically.”* Another partner commented on the efficiency of interprofessional communication stating, *"receiving doctors should be able to read the clinical summary and know exactly where to start."*

#### 3.2.2 Time burden

Clinical partners noted there were no negative effects of the program design except for the additional time required during visits to address transition needs. They reported PEAKS visits took approximately 10–15 minutes longer than a traditional follow up visit. This included time spent reviewing the READDY survey, discussing the transition process or policy, and the additional documentation required for all program elements. One clinical partner noted, *“We may get a little behind in the check-in process,”* referring to patient completion of the READDY survey which often interrupted the scheduled start time with the provider. Due to the volume of education modules covered in fewer visits, clinical partners referred to catch up visits as *“time consuming.”* In addition, the transition-focused education across all visits was noted to add at least an additional 5 minutes to time spent in the room with patients.

#### 3.2.3 Identification of patient knowledge gaps

The ability to better identify and correct patient knowledge deficits in a timely fashion prior to discharge was also reported by several clinical partners. One partner commented, *"Do you have any questions? - that is so vague. Now, we use tools and processes to really figure out where the knowledge and skill gaps lie."* Another commented, *"We are no longer assuming that they are more prepared than they actually are; we can take some additional time to fill those gaps before they leave - that is time well-spent."*

### 3.3 Balancing measure

The theme, value despite time-burden, was identified from comments related to the acceptability of the program. Clinical partners verbalized satisfaction with individual program elements despite the additional time added to clinic visits. They found the program to be “*relevant*” and “*well-received [by patients]."* In addition, partners indicated that the program, “*allowed us to sever ties gracefully.”* Although visit length did increase, they deemed the various program components as worthwhile. A clinical partner commented, *"The added time often comes from longer discussions based on identified deficits - but it's not bad time - it's valuable time and it should be prioritized.”* Another commented, *"It provides an opportunity to follow a specific program pathway and truly spend time engaging in focused transition."*

## 4 Discussion

### 4.1 Summary

Despite eleven years passing since the release of the ADA’s call for a focus on healthcare transition, organized care processes are still lacking.^25^ At this institution, there was a new system-wide focus on transitional care, as well as vested clinical partners who championed the improvement of transition practices. Through a collaborative quality improvement approach, the program was designed to leverage the EHR platform, incorporate diabetes-specific transition recommendations, and tailor the program to the unique needs of the clinic following a standardized health care transition program. A fully integrated T1D transition program is now in place in this pediatric endocrinology setting and clinical partners have deemed the PEAKS program design to be both acceptable and practical.

#### 4.1.1 Strengths of the program

There were several strengths of this quality improvement project which established a formal transition process. First, a standardized transition framework was used to guide program design. The Got Transition® *Transitioning Youth to and Adult Health Care Clinician Implementation Guide*^*21*^ was used as the structure and tailored to the individual clinic’s needs and population. Additionally, the program design spread the tasks of health care transition among the clinic providers, which was important in this project, as the institution was unable to allocate funding for a health care transition coordinator. A second key strength of the program is alignment with the ADA’s recommendations for transition of adolescents with T1D to adult care. For example, the education deemed necessary for adolescents with T1D to achieve independent self-care were all included in the PEAKS modules over the course of the program. Finally, integration into the EHR allowed the program to be seamlessly adopted into the existing workflow of the clinic. For example, documentation templates and automated billing codes allowed the additional work of transitional care to be provided in an efficient manner.

### 4.2 Interpretation

Unintended consequences of program implementation were mostly centered on adding time and technology requirements to appointments for patients, roomers (nurses and medical assistants), certified diabetes educators, and physician providers. For example, patients needed to have a working patient-portal account and access to the internet during the appointment to complete the READDY survey. If they did not have an account or experienced internet issues, the survey had to be administered on paper and answers had to be manually entered into the EHR by the project leader or diabetes educator, requiring additional time and potentially leading to missing data.

The "catch-up" visits were another area of identified time burden. However, the "catch-up" visits were deemed necessary from an ethical standpoint. After the need for an improved transition process was identified in this setting, the knowledge and skill deficits in those older than enrollment age, could not simply be ignored. This particular challenge should be self-limiting as the PEAKS program continues to be offered and future patients are enrolled at the designated 16-year-old visit.

A final confounding factor that added to time burden is related to the project setting. The academic medical center that served as the project site hosts multiple pediatric residency programs. This led to time delays as multiple learners and providers were sequentially involved in the patient visit.

As other transition programs are developed within the system, a designated Epic analyst would enhance efficiency of the program-build process. An Epic analyst was essential to integrating the PEAKS program within the EHR. Since Epic analysts at this institution work on multiple projects simultaneously, time delays occurred as the project leader was unable to build or change program components independently when an analyst was unavailable. In addition to a dedicated analyst, regularly scheduled meetings to accomplish the work of program integration would enhance efficiency.

While informal feedback was solicited from some adolescents and families/caregivers during early stages of design and development, future transition program development in other specialties should include more formalized membership on the partnership team from these populations. As the PEAKS program continues, collection and evaluation of Got Transition's youth/young adult and parent/caregiver feedback surveys^26^, ^27^ during the 6-month follow up period is necessary to ensure that the program is meeting the needs of the patient population served. Another important next step will include continued program evaluation focused on the effectiveness of the program in relation to patient outcomes. One way to achieve this will be comparison of the READDY assessment results between PEAKS enrollment and PEAKS discharge visits. Other measures might include tracking disease management markers and attendance at the first adult appointment. Additionally, development of an advisory council that includes graduates of the PEAKS program will be imperative for the sustainability and growth of the program.

### 4.3 Limitations

This quality improvement project had several limitations. Although informal feedback was solicited from adolescents and parents/caregivers, they were not included as formal partners in the adaptation and integration phases of the PEAKS program development. As data continue to be collected from the 6-month follow up surveys, feedback from adolescents and parents/caregivers will be available to inform future changes to the PEAKS program. The generalizability of our findings is limited. The PEAKS program was designed and implemented to meet the specific needs of this particular clinic and all program elements may not be practical and acceptable in other clinics. For example, the program was built within Epic and integration into another EHR may require significant adaptation. Additionally, smaller health care systems may not have the information technology support necessary for the program build. Lastly, further program evaluation will need to consider patient outcome measures such as knowledge and skill attainment, and ultimately disease management markers.

## 5 Conclusions and future directions

The PEAKS program could be expanded in several ways. The program is a feasible way to offer best-practice diabetes management to emerging adults with T1D. As more patients complete the PEAKS program, an advisory board that includes people who have completed the program will be possible. Hiring of a healthcare transition coordinator would enhance efficiency of program delivery and further streamline clinic workflow related to transition. PEAKS can serve as a model for development of transitional care programs in other pediatric sub-specialty clinics in this local academic healthcare center and across the healthcare system.

The development of this transition program incorporated clinical partner feedback, used a quality improvement approach, and was tailored to include ADA recommendations and the unique needs of the clinic. The PEAKS program is practical and acceptable to the providers in the clinic, despite the time burden. The program is an example of incorporating organized transitional care processes, and facilitating a team-based approach, interdisciplinary communication, and patient-centered care.

## Article submission ethical statement

We have reviewed the Ethics in Publishing content for authors and have no ethical concerns, issues, or problems to reports.

## Funding

This research did not receive any specific grant from funding agencies in the public, commercial or not-for-profit sectors.

## CRediT authorship contribution statement

**Emily Barnes:** Writing – review & editing, Methodology, Formal analysis, Conceptualization. **Billie Vance:** Writing – review & editing, Visualization, Methodology, Formal analysis, Conceptualization. **Elizabeth Ann Minchau:** Writing – review & editing, Writing – original draft, Visualization, Methodology, Formal analysis, Conceptualization.

## Declaration of Competing Interest

The authors declare that they have no known competing financial interests or personal relationships that could have appeared to influence the work reported in this paper.

## Data Availability

No data was used for the research described in the article.
